# GANAB-Associated Severe Autosomal Dominant Polycystic Kidney Disease in an 18-Year-Old Female: A Case Report

**DOI:** 10.7759/cureus.79498

**Published:** 2025-02-23

**Authors:** Gautam Agrawal, Bhawna Agarwal, Anjana Chandrasekhara Pillai, Kiran Kuriakose

**Affiliations:** 1 Nephrology, Independence Health System, Greensburg, USA; 2 Internal Medicine, University of Pittsburgh Medical Center McKeesport Hospital, McKeesport, USA; 3 Internal Medicine, Saint Clair Hospital, Pittsburgh, USA; 4 Internal Medicine, University of Pittsburgh Medical Center, Pittsburgh, USA

**Keywords:** adpkd, autosomal-dominant polycystic kidney disease, bilateral renal cysts, genetic renal diseases, hereditary renal disease, renal cysts, tolvaptan treatment

## Abstract

Autosomal dominant polycystic kidney disease (ADPKD) is a hereditary disorder, characterized by the formation of multiple cysts in the kidneys, leading to progressive kidney enlargement and, eventually, renal failure. It is most frequently associated with PKD1 or PKD2 mutations, although rare variants, such as the GANAB gene, are also associated, but they present as a milder renal phenotype. ADPKD patients often present with renal manifestations, such as hypertension, abdominal pain, hematuria, or urinary tract infections, along with extrarenal manifestations, such as liver cysts, heart valve disease, and cerebral aneurysms. ADPKD is usually diagnosed in the fourth or fifth decade of life.

This case report discusses the clinical presentation, diagnostic approach, and management of an 18-year-old female patient with no known first-degree family history of ADPKD, who presented with hypertension and bilateral renal cysts on ultrasound. The diagnosis was confirmed by imaging studies and genetic testing. The GANAB gene mutation found in this patient is typically associated with mild kidney disease; however, according to the Mayo Clinic Imaging Classification (MIC) for ADPKD, our patient falls under Classification 1E, which is predictive of rapid progression to end-stage renal disease (ESRD). It highlights the challenges in treating young patients with ADPKD, given the limited studies available for managing this progressive disease in the young population. This case questions the assumption that GANAB-associated ADPKD progresses in a mild manner. Clinicians should prioritize vigilant monitoring and a multidisciplinary approach for young patients with high-risk imaging characteristics, regardless of their genetic findings.

## Introduction

Autosomal dominant polycystic kidney disease (ADPKD) is the most common hereditary kidney disease [1], characterized by the progressive development of multiple fluid-filled cysts within the kidneys, leading to kidney enlargement and eventual organ failure. ADPKD is the fourth most common cause of end-stage renal disease (ESRD), highlighting the disease burden [2]. Renal complications associated with ADPKD include hypertension, kidney stones, hematuria, proteinuria, and infections, while extrarenal complications include liver cysts, heart valve disease, and cerebral aneurysms. Abdominal pain is a common symptom of ADPKD, often leading to depression and affecting quality of life [3]. Approximately 50% of individuals with ADPKD will develop ESRD by age 60 [4]. Risk factors for disease progression include younger age at diagnosis, large kidney volume, rapid cyst growth, hypertension, male gender, and hematuria.

The diagnosis of ADPKD is confirmed with kidney imaging criteria, along with either a first-degree relative with ADPKD or genetic testing [4]. Common genes associated with ADPKD are PKD1 or PKD2, while less common genes include ALG5, ALG9, DNAJB11, GANAB, and IFT140 [4]. Patients with GANAB or ALG9 mutations usually have mild disease that rarely progresses to ESRD [5]. Management of ADPKD focuses on controlling symptoms, managing blood pressure, and slowing progression to ESRD.

This case report details a young female patient diagnosed with ADPKD at the age of 18, with no known first-degree family history. The diagnosis was confirmed with imaging and genetic testing, revealing a GANAB gene mutation - likely pathogenic, which typically presents with mild kidney disease. However, in our patient, the disease was found to be severe, as classified by the Mayo Clinic Imaging Classification (MIC) for ADPKD [6]. The case highlights the need for refining ADPKD guidelines for management in adolescents, particularly with high-risk imaging features despite reassuring genotypes.

## Case presentation

This is an 18-year-old patient who initially presented to a primary care physician with abdominal discomfort, prompting an ultrasound of the abdomen. The ultrasound revealed gallstones and multiple bilateral renal cysts. Consequently, she underwent an uncomplicated cholecystectomy. She was referred to nephrology for further evaluation due to concern for polycystic kidney disease (PKD). Although she had no first-degree family history of ADPKD, a distant relative had this disease.

Her physical exam was unremarkable. She was 4 feet 11 inches (1.5 meters) tall and weighed 155 lbs. Her lungs were clear, and her heart rate was normal with a regular rhythm. She appeared euvolemic with no peripheral edema. She was hypertensive, with a blood pressure of 138/84 mmHg. Her blood work was unremarkable and showed normal renal function and liver function tests, as detailed in Table 1.

**Table 1:** Laboratory values - renal function and liver function tests

Lab Test	Results	Reference Range	Units
Hemoglobin	13.3	11.7-15.8	gm/dL
Sodium	139	136-145	mEq/L
Potassium	4	3.5-5	mEq/L
Blood Urea Nitrogen (BUN)	10	7-25	mg/dL
Creatinine	0.7	0.6-1.2	mg/dL
Calcium	9.8	8.6-10.3	mg/dL
Albumin	4.7	3.5-5.7	gm/dL
Alkaline Phosphatase (Alk Phos)	75	34-104	unit/L
Serum Glutamate-Oxaloacetate Transaminase (SGOT)	19	13-39	unit/L
Alanine Aminotransferase (ALT)	19	7-52	unit/L
Total Bilirubin (Bili total)	0.4	0.3-1	mg/dL

The urinalysis was clear, with negative protein, no blood or casts, and no white blood cells (WBCs) or red blood cells (RBCs).

Imaging studies

A magnetic resonance imaging (MRI) was performed, confirming multiple renal cysts consistent with PKD. No liver abnormalities were detected. Table 2 shows the length, width, and depth measurements of the right and left kidneys in our patient, which show significant enlargement of the kidneys bilaterally compared to the normal reference age.

**Table 2:** Length, width, and depth of right and left kidney

Measurement	Right Kidney	Left Kidney	Reference Range
Coronal Length (cm)	14.1	14.2	9-13 cm
Width (cm)	7.9	8.85	3-5 cm
Depth (cm)	8.15	8.2	3-4 cm

**Figure 1:**
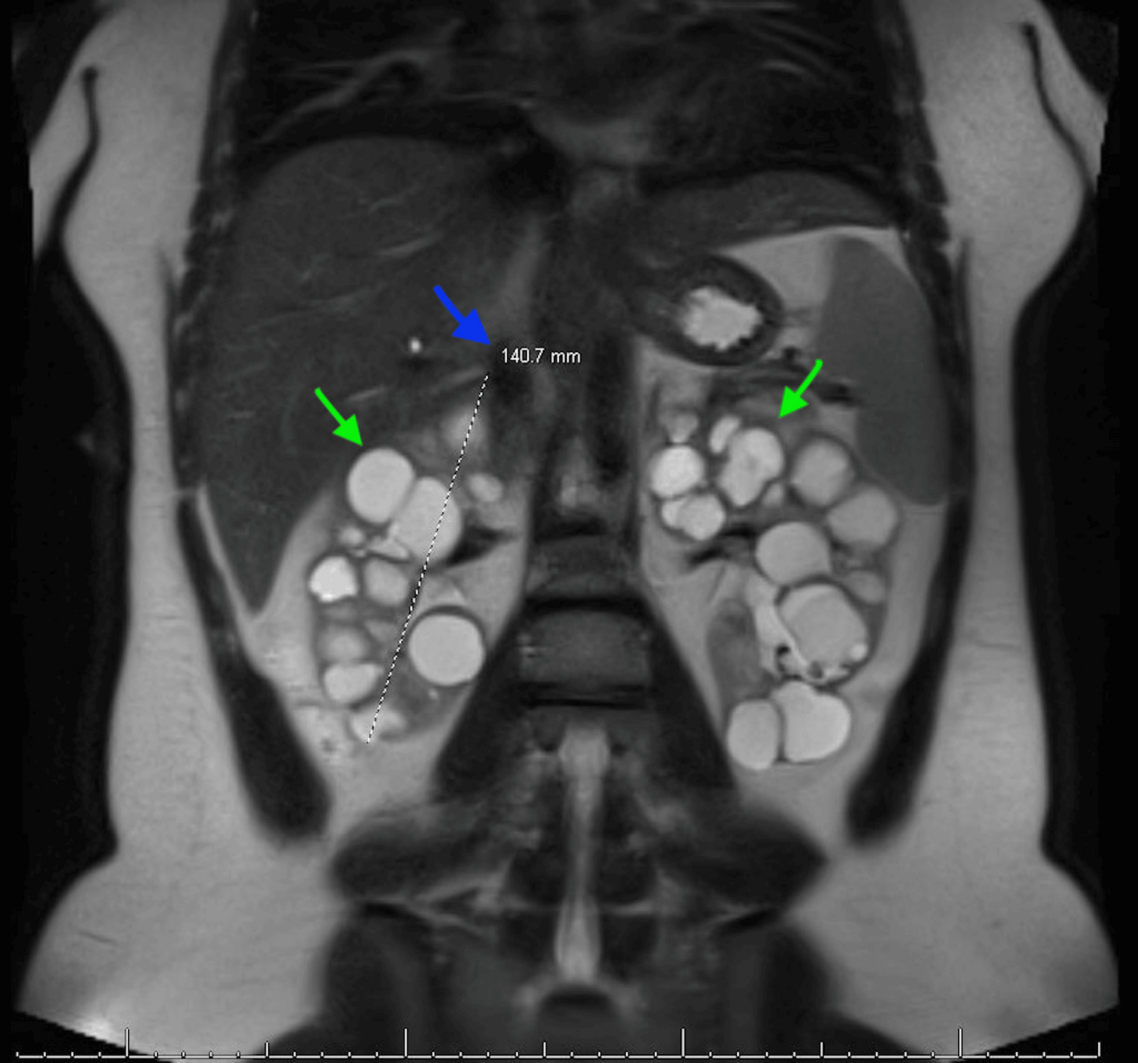
Coronal section indicating bilaterally enlarged kidneys with renal cysts, and measurement of the right kidney length Blue arrow indicating right coronal length of 140.7 mm; Green arrows indicating multiple renal cysts bilaterally

**Figure 2:**
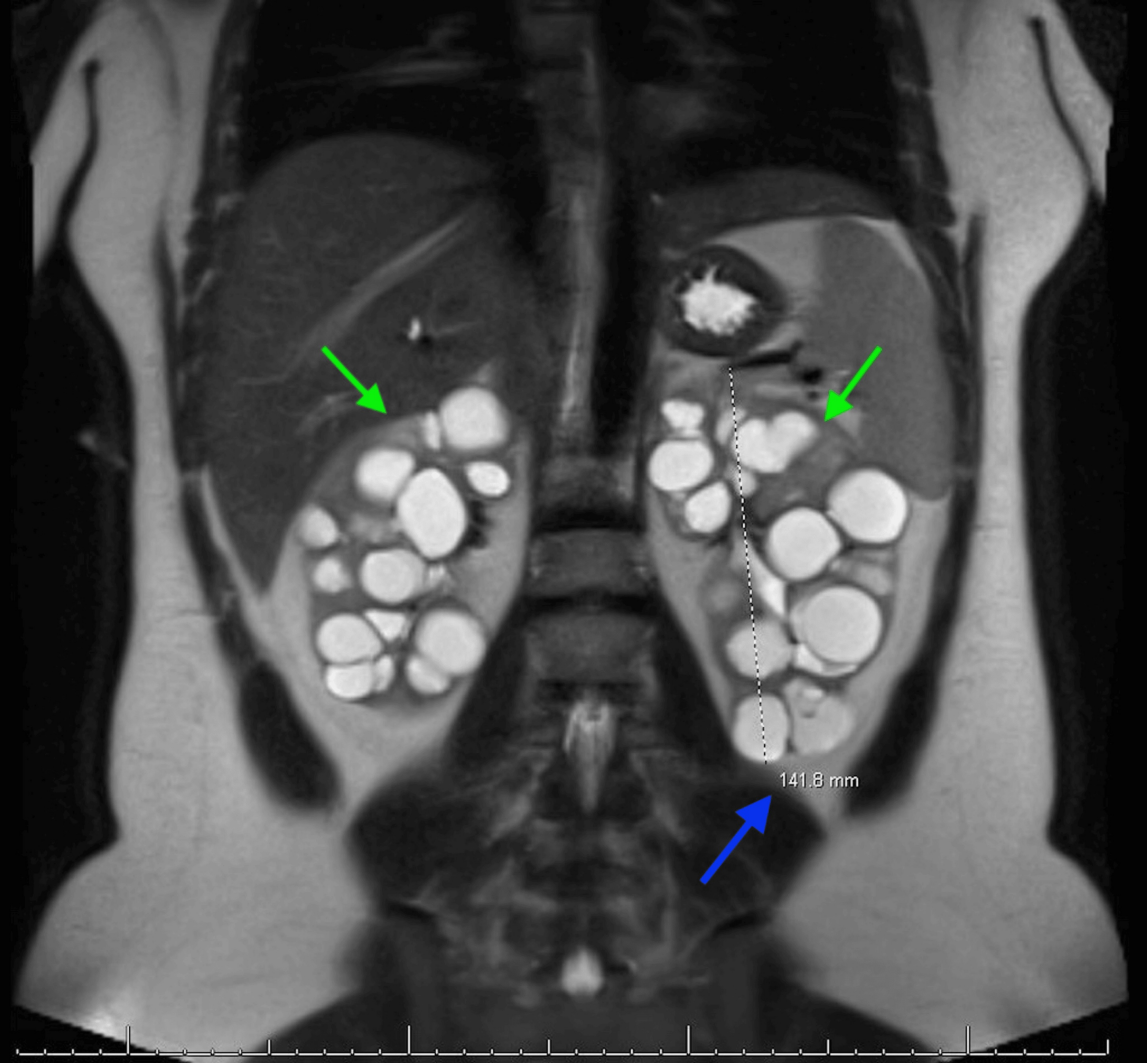
Coronal section indicating bilaterally enlarged kidneys with renal cysts, and measurement of left kidney length Blue arrow indicating left kidney length of 142 mm; Green arrows indicating renal cysts bilaterally

**Figure 3:**
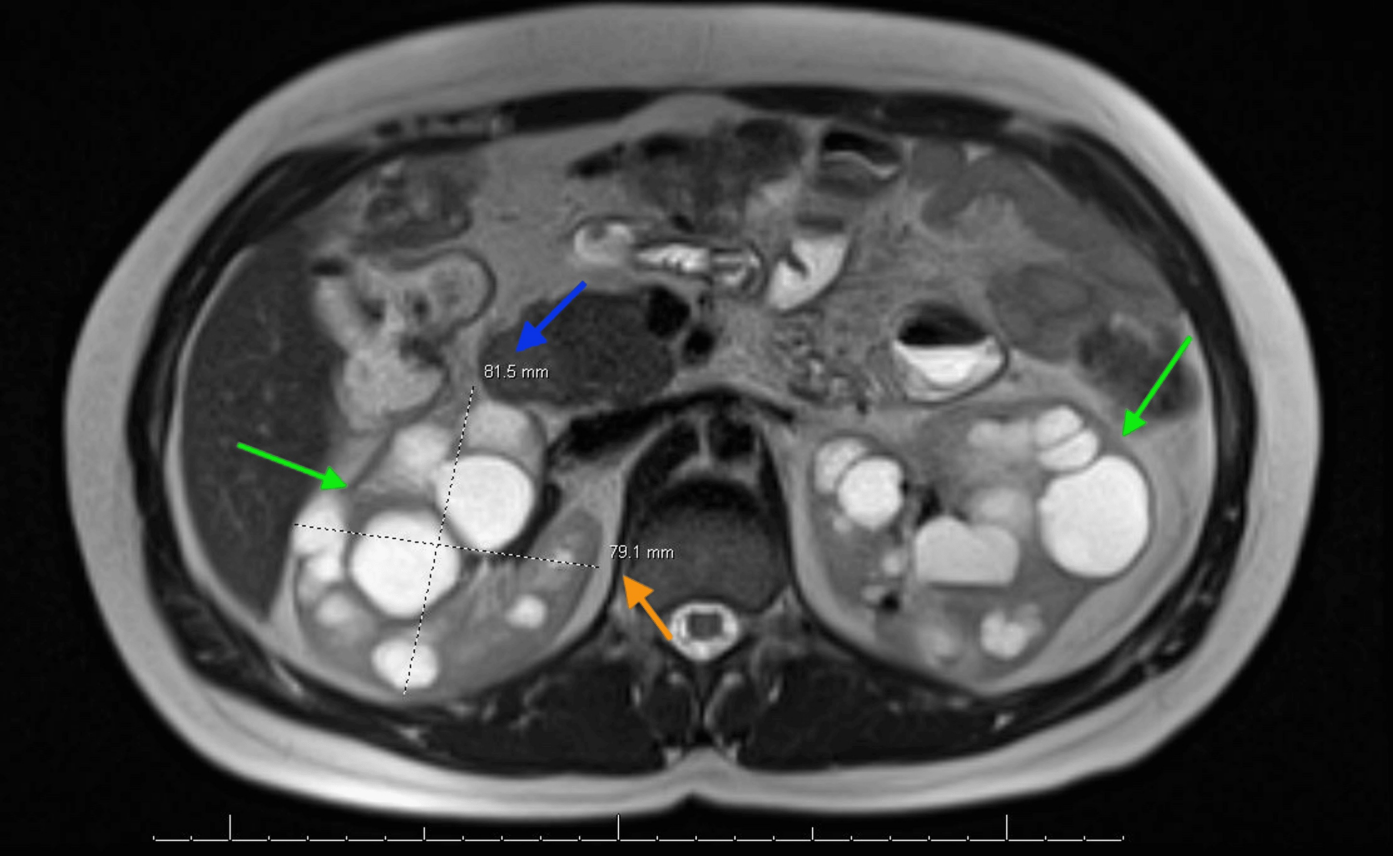
Bilateral renal cysts, along with measurements of depth and width of the right kidney Blue arrow indicating depth measurement of 81.5 mm; Orange arrow indicating width measurement of 79.1 mm; Green arrows indicating bilateral renal cysts

**Figure 4:**
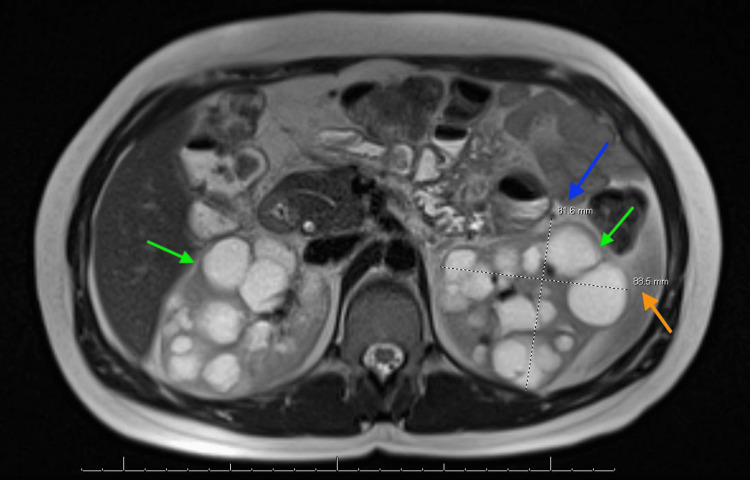
Bilateral enlarged kidneys with renal cysts, along with measurement of depth and width of the left kidney Blue arrow indicating depth measurement of 81.6 mm; Orange arrow indicating width measurement of 88.5 mm; Green arrows indicating bilateral renal cysts

Below are the MRI images of the patient, which show severely enlarged kidneys with multiple renal cysts, confirming the diagnosis of PKD. Figure 1 shows a coronal section indicating bilaterally enlarged kidneys with multiple renal cysts, with a blue arrow indicating the length measurement of the right kidney. Figure 2 shows a coronal section indicating bilaterally enlarged kidneys with multiple renal cysts, with a blue arrow indicating the length measurement of the left kidney. Figure 3 indicates bilateral polycystic kidney enlargement, with the blue arrow indicating depth and the orange arrow indicating the width measurement of the right kidney. Figure 4 indicates bilateral polycystic kidney enlargement, with the blue arrow indicating depth and the orange arrow indicating the width measurement of the left kidney. Green arrows in all figures indicate bilaterally enlarged kidneys with multiple renal cysts.

Total kidney volume (TKV) is calculated using the ellipsoid volume formula, which is: $V = \left(\frac{6}{\pi}\right) \times (L \times W \times D)$, where L = Length, W = Width, and D = Depth. The right kidney volume, based on the above formula, was 478.3 mL, and the left kidney volume was 542.6 mL. TKV is the sum of the right and left kidney volumes, which equals 1020.9 mL. The height-adjusted TKV (htTKV) = TKV/Height, which came back to 680.6 mL/min (1020.9 mL/1.5 m = 680.6 mL/m). Based on the htTKV and age, the patient falls under Classification 1E of the MIC for ADPKD [6]. Class 1E indicates an estimated kidney growth rate of >6% yearly, with a high risk for estimated glomerular filtration rate (eGFR) decline.

Genetic testing revealed a variant in the GANAB gene, associated with polycystic kidney and/or polycystic liver disease. She was started on lisinopril for better blood pressure control, with a target blood pressure of <110/75 mmHg, as recommended in this population [3]. The patient was extensively counseled by genetic counselors and nephrologists. Given her high TKV and risk classification, she was started on tolvaptan. The side effects, including liver toxicity, nocturia, and polyuria, were discussed in detail with her, and she agreed to start the medication. Liver and renal function were to be monitored closely every two weeks for one month, and then monthly for 18 months. Over the 10 months she has been on tolvaptan, she has tolerated it well, and her renal and liver function tests have remained stable.

## Discussion

This case highlights the sequence of diagnostic tests, use of imaging, and genetic testing to confirm the diagnosis and management of ADPKD in a young patient with severe disease, according to the MIC criteria. ADPKD is typically diagnosed in the fourth or fifth decade of life. The GANAB gene mutation is generally associated with mild disease, but our patient was found to have severe disease. It is crucial to carefully assess the risk of disease progression in ADPKD patients to determine the need for early interventions aimed at slowing progression, after evaluating the risks and benefits of such interventions [7]. The MIC criteria for ADPKD are used to predict disease progression based on htTKV [7]. Patients in MIC classes 1C-1E are at risk of rapid progression to ESRD and benefit from early intervention with disease-modifying treatment, such as tolvaptan [3].

Tolvaptan is a vasopressin V2 receptor (V2R) antagonist that blocks vasopressin signaling, a key driver of cyst growth in ADPKD. It is an important treatment option for patients with severe disease. Tolvaptan has been shown to reduce annual eGFR loss by approximately 1 mL/min/1.73 m² in two important trials, TEMPO 3:4 (Tolvaptan Efficacy and Safety in the Management of ADPKD and its Outcomes) [8] and REPRISE (Replicating Evidence of Preserved Renal Function: An Investigation of Tolvaptan Safety and Efficacy in ADPKD) [9,3]. Tolvaptan is the only treatment proven to slow the progression of ADPKD, as measured by changes in eGFR and TKV [10].

There is limited data on the clinical presentation of young patients with ADPKD. A Spanish study of young adults (ages 18-30 years) with ADPKD found that 36.8% of patients aged 25-30 years had hypertension, despite normal eGFR. Young adults with ADPKD experience significant morbidity related to the condition. This highlights the importance of comprehensive assessment and management of young ADPKD patients, ideally before any decline in eGFR occurs [11]. For patients under 50 years old and with eGFR >60 mL/min/1.73 m², a blood pressure goal of ≤110/75 mmHg is recommended. In the HALT (Halting Progression of Polycystic Kidney Disease) PKD A trial, patients who were treated to reach a lower blood pressure target experienced slower growth in TKV compared to those who were treated to a standard blood pressure target [3].

## Conclusions

ADPKD is a hereditary disorder that typically presents in the fourth or fifth decade of life and is more common in patients with a known first-degree family history of the condition. This case highlights the presentation of severe ADPKD in a young patient with no known first-degree family history of ADPKD. She had a GANAB gene mutation, a gene tied to mild kidney disease but atypically developing rapidly progressing cysts (MIC 1E). This warranted early intervention, including strict blood pressure control and the use of tolvaptan to decrease the risk of progression to ESRD. Further research is required to gain a deeper understanding of the clinical progression of ADPKD in young patients and to evaluate the safety of treatments in adolescents.
